# Glycine-alanine dipeptide repeat protein contributes to toxicity in a zebrafish model of *C9orf72* associated neurodegeneration

**DOI:** 10.1186/s13024-016-0146-8

**Published:** 2017-01-14

**Authors:** Yu Ohki, Andrea Wenninger-Weinzierl, Alexander Hruscha, Kazuhide Asakawa, Koichi Kawakami, Christian Haass, Dieter Edbauer, Bettina Schmid

**Affiliations:** 1 German Center for Neurodegenerative Diseases (DZNE), Feodor-Lynen-Str.17, 81377 Munich, Germany; 2 Biomedical Center, Biochemistry, Ludwig-Maximilians University Munich, Feodor-Lynen-Str.17, 81377 Munich, Germany; 3 Division of Molecular and Developmental Biology, National Institute of Genetics, Mishima, Shizuoka 411-8540 Japan; 4 Munich Cluster for Systems Neurology (SyNergy), Feodor-Lynen-Str.17, 81377 Munich, Germany

**Keywords:** Zebrafish, *C9orf72*, poly-GA toxicity

## Abstract

**Background:**

The most frequent genetic cause of frontotemporal lobar degeneration (FTLD) and amyotrophic lateral sclerosis (ALS) is the expansion of a GGGGCC hexanucleotide repeat in a non-coding region of the chromosome 9 open reading frame 72 (*C9orf72)* locus. The pathological hallmarks observed in *C9orf72* repeat expansion carriers are the formation of RNA foci and deposition of dipeptide repeat (DPR) proteins derived from repeat associated non-ATG (RAN) translation. Currently, it is unclear whether formation of RNA foci, DPR translation products, or partial loss of C9orf72 predominantly drive neurotoxicity in vivo. By using a transgenic approach in zebrafish we address if the most frequently found DPR in human ALS/FTLD brain, the poly-Gly-Ala (poly-GA) protein, is toxic in vivo.

**Method:**

We generated several transgenic UAS responder lines that express either 80 repeats of GGGGCC alone, or together with a translation initiation ATG codon forcing the translation of GA80-GFP protein upon crossing to a Gal4 driver. The GGGGCC repeat and GA80 were fused to green fluorescent protein (GFP) lacking a start codon to monitor protein translation by GFP fluorescence.

**Results:**

Zebrafish transgenic for the GGGGCC repeat lacking an ATG codon showed very mild toxicity in the absence of poly-GA. However, strong toxicity was induced upon ATG initiated expression of poly-GA, which was rescued by injection of an antisense morpholino interfering with start codon dependent poly-GA translation. This morpholino only interferes with GA80-GFP translation without affecting repeat transcription, indicating that the toxicity is derived from GA80-GFP.

**Conclusion:**

These novel transgenic *C9orf72* associated repeat zebrafish models demonstrate poly-GA toxicity in zebrafish. Reduction of poly-GA protein rescues toxicity validating this therapeutic approach to treat *C9orf72* repeat expansion carriers. These novel animal models provide a valuable tool for drug discovery to reduce DPR associated toxicity in ALS/FTLD patients with *C9orf72* repeat expansions.

**Electronic supplementary material:**

The online version of this article (doi:10.1186/s13024-016-0146-8) contains supplementary material, which is available to authorized users.

## Background

Expansion of the GGGGCC hexanucleotide repeat in the *C9orf72* intronic region was recently identified as a cause for amyotrophic lateral sclerosis (ALS) and frontotemporal lobar degeneration (FTLD) [1–3]. This repeat expansion is observed in around 40% of familial and 7% of sporadic cases of ALS and 25% of familial and 6% of sporadic cases of FTLD [4]. Affected patients have hundreds to several thousands of repeats, while healthy individuals generally have 2 to 23 repeats [1–3, 5]. The expanded repeat RNA is transcribed and accumulates in RNA foci, which have been detected in brain tissue, lymphoblasts, as well as fibroblasts derived from patients with the *C9orf72* associated repeat expansion [6]. This long repeat RNA transcript can sequester RNA binding proteins, including heterogeneous nuclear ribonucleoprotein A3 (hnRNPA3), hnRNPH, and nucleolin, and can lead to mis-regulation of RNA splicing [7–9]. Interestingly, despite the absence of an ATG start codon, the repeat RNA is further subjected to unconventional **r**epeat **a**ssociated **n**on-ATG (RAN) translation [7, 9–11] resulting in dipeptide repeat proteins (DPRs) of Gly-Ala (poly-GA), Gly-Arg (poly-GR) and Gly-Pro (poly-GP) and additional Gly-Pro (poly-GP), Pro-Ala (poly-PA) and Pro-Arg (poly-PR) from the transcribed antisense strand. The DPRs form cytosolic coaggregates with p62 in the brains of patients with *C9orf72* repeat expansions [12–15] and have been shown to interfere with RNA metabolism and coaggregate with other proteins [16–20]. Additionally, interference of DPRs with nucleocytoplasmic transport has been identified independently in different model systems by unbiased genetic screens [21–25].

Three pathomechanisms have been postulated in *C9orf72* repeat expansion carriers, which are not mutually exclusive and most likely act in combination. First, haploinsufficiency due to reduced transcript levels of *C9orf72*. Second, toxicity of RNA foci by sequestration of important RNA binding proteins and disturbed RNA homeostasis. Third, toxicity of RAN translation products.

We set out to generate transgenic zebrafish lines with expanded GGGGCC repeats and poly-GA as a vertebrate animal model to address their contribution to toxicity. We generated two transgenic lines expressing 80 repeats of the GGGGCC sequence (ggggcc80) and two lines with the translation initiation codon ATG in front of the 80xGGGGCC repeat sequence driving expression of poly-GA protein fused to green fluorescent protein (GA80-GFP). We chose poly-GA since it is the most abundant DPR species found in patients with *C9orf72* repeat expansions [13, 26]. The transgenic zebrafish models with 80 repeats reproduced key pathological features, such as RNA foci, however RAN translation was not detectable. Transgenic zebrafish with 80 repeats of GGGGCC only showed minor toxicity (mild pericardial edema), which was greatly increased when we forced expression of poly-GA by 80 GGGGCC repeats with an ATG translational start codon in the GA frame (severe pericardial edema). By blocking poly-GA translation by an antisense approach, we show that the phenotypes can be partially rescued, demonstrating that poly-GA is toxic in vivo and that targeting poly-DPRs might be a useful therapeutic strategy for *C9orf72* repeat expansion carriers.

## Results

### Generation of a transgenic zebrafish model of *C9orf72* repeat expansion disease

**Fig. 1:**
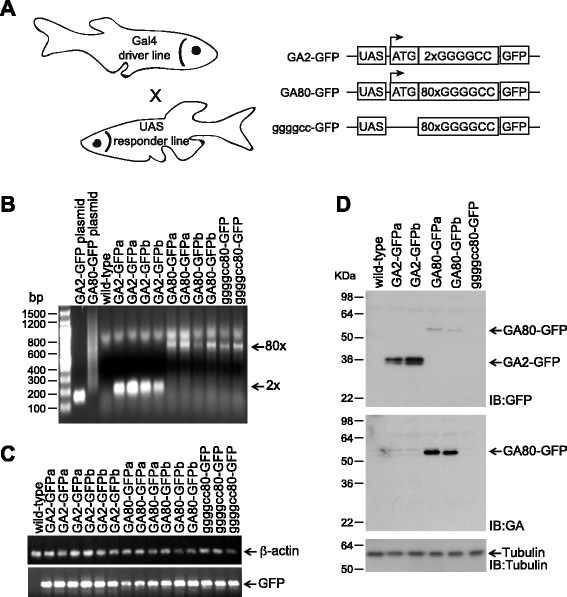
Generation of transgenic zebrafish model of *C9ORF72* repeat expansion disease. **a** Schematic representation of Gal4 driver line zebrafish crossed to a UAS responder transgenic zebrafish to generate embryos that express a transgene under the control of the UAS. Schematic representation of the responder constructs used for the generation of transgenic zebrafish. **b** Genotyping by PCR of 1 dpf embryos. pCS2 + 2xGGGGGCC and pCS2 + 80XGGGGCC constructs were used as standards for GA2-GFP and GA80-GFP in lane 2 and 3. Positions of 2xGGGGCC and 80xGGGGCC repeats are indicated by arrows. **c** Semi-quantitative RT-PCR analyses for the wild-type, GA2-GFPa/b, GA80-GFPa/b and ggggcc80-GFP zebrafish. Note that all transgenic lines showed similar expression level at 4 dpf embryos. **d** Immunoblotting of wild-type, GA2-GFPa/b, GA80-GFPa/b and ggggcc80-GFP with antibodies as indicated with embryonic lysates of 4 dpf old embryos

We generated several transgenic zebrafish UAS-based responder lines expressing either 2 or 80 repeats with an ATG (Tg(UAS:ATG-2xGGGGCC-GFP) and Tg(UAS:ATG-80xGGGGCC-GFP)) in the GA reading frame as well as 80 GGGGGCC repeats without ATG (Tg(UAS:80xGGGGCC-GFP)) fused to GFP (Fig. 1a). We generated these lines by Tol2 mediated transposition into the zebrafish genome [27]. We confirmed successful germline transmission of the transgenes by PCR-based genotyping. The 80xGGGGCC repeat sequence was unstable and changes in repeat length were frequently observed in the F1 generation (data not shown). We selected stable transgenic lines with 80xGGGGCC repeats and confirmed the repeat length by PCR (Fig. 1b). To exclude potential toxicity mediated by the transgene integration site, we selected 2 independent lines (a and b) for Tg(UAS:ATG-2xGGGGCC-GFP) and Tg(UAS:ATG-80xGGGGCC-GFP), which we will refer to as GA2-GFPa/b and GA80-GFPa/b respectively. Comparable mRNA expression levels of the respective transgene were confirmed by semi-quantitative reverse transcription PCR (RT-PCR) upon crossing the responder lines to the gene trap Gal4 driver line SAGFF73A [28]. This line drives expression of high level of Gal4 ubiquitously at early embryonic stages and was used in all experiments to drive transgene expression of the respective UAS driver lines. All transgenic lines showed comparable RNA expression levels at 4 days post fertilization (dpf) (Fig. 1c). Additionally, we confirmed the expression of GA80-GFP protein by immunoblotting at 4 dpf (Fig. 1d). The GA2-GFPa/b larvae showed 7.2 fold higher GFP protein expression levels compared to the GA80-GFPa/b larvae (mean of line a and b). This suggests that despite nearly equal mRNA levels, less poly-GA is translated in the GA80-GFP fish than the GA2-GFP fish. A poly-GA specific antibody also detected the GA80-GFP protein (Fig. 1d), but failed to detect GA2-GFP, most likely because two GA repeats are too short to be recognized by the antibody.

Tg(UAS:80xGGGGCC-GFP) fish expressing repeat RNA without a start codon were generated and fish with a stable length of 80 repeats were selected by PCR (Fig. 1c). This line will be referred to as ggggcc80-GFP. Although the expression level of mRNA is similar to those of GA2-GFP and GA80-GFP expressing fish, poly-GA peptides derived from RAN translation were not detectable by Western blotting in the ggggcc80-GFP fish at 4 dpf (Fig. 1d). We also failed to detect poly-GR and poly-GP (data not shown). Thus, if RAN translation occurs in the ggggcc80-GFP line, it is below the detection limit of our specific antibodies.

### RNA derived from GA80-GFP and ggggcc80-GFP lead to RNA foci formation

**Fig. 2:**
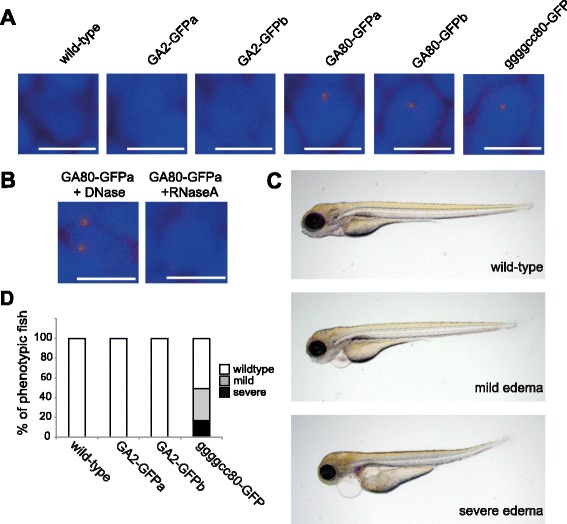
RNA foci formation in transgenic zebrafish. **a**, **b** Cy3-labeled in situ probe detected dot-like structures in spinal cord in GA80-GFPa/b and ggggcc80-GFP zebrafish. **b** Foci were only detected in GA80-GFP and ggggcc-GFP fish whereas no foci were detected in wild-type and GA2-GFPa/b fish at 28 hpf. GA80-GFPa zebrafish were treated with RNaseA or DNase. Scale bar 10 μm. **c** Pericardial edema phenotype observed in ggggcc80-GFP zebrafish at 4 dpf. Phenotypic features are classified as wild-type, mild edema, and severe edema. **d** The average percentages of phenotypic fish of the three different phenotypic classes at 4 dpf are indicated in the bargraph (at least three independent clutches were analyzed with *n* ≥ 14)

To test if pathological hallmarks of *C9orf72* repeat expansion disease are found in transgenic zebrafish with an expanded GGGGCC repeat, we analyzed RNA foci formation at 28 h post fertilization (hpf). GA80-GFP as well as ggggcc80-GFP zebrafish showed RNA foci in spinal cord neurons by in situ hybridization, whereas wild-type and GA2-GFP zebrafish did not show RNA foci (Fig. 2a and Additional file 1: Figure S1). We further confirmed that the RNA foci are sensitive to RNaseA but not to DNase treatment, confirming the RNA nature of the foci (Fig. 2b and Additional file 1 Figure S1).

### ggggcc80-GFP expression displays mild toxicity in zebrafish

We next analyzed ggggcc80-GFP transgenic fish for signs of toxicity. ggggcc80-GFP larvae showed a pericardial edema phenotype at 4 dpf. We categorized the phenotypes based on their severity in three groups: 1. wild-type, 2. mild edema and 3. severe pericardial edema as shown in Fig. 2c. Of the ggggcc80-GFP larvae 50.2 ± 13.7% (mean ± SD) were unaffected, 32.2 ± 8.2% had a mild pericardial edema and 17.2 ± 11.6% had a severe pericardial edema at 4 dpf (Fig. 2d). The edema phenotype precluded inflation of the swim bladder, inability to feed independently, and death during early larval stages. No abnormal phenotype was detectable in GA2-GFP larvae, suggesting that the expanded GGGGCC repeat causes the mild toxicity in the ggggcc80-GFP fish.

### GA80-GFP protein expression is highly toxic in zebrafish

**Fig. 3:**
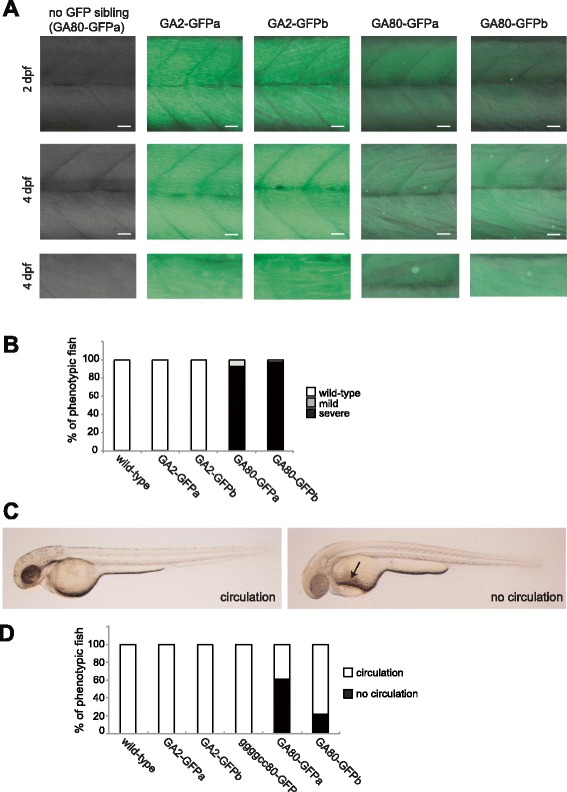
Poly-GA protein elicits a toxic phenotype. **a** In vivo image of GA-GFP polypeptides at 2 and 4 dpf. Genotypes as indicated. No GFP sibling (GA80-GFP) refers to a sibling from a cross between a Gal4 driver and a UAS GA80-GFP responder fish that is GFP negative, and hence is either negative for the driver or the responder construct, or both constructs. GFP fluorescent images shown are merged with DIC pictures. Lowest panel is a magnification of the middle panel at 4 dpf. Lateral views of the trunk musculature. Scale bar 20 μm. **b** A strong pericardial edema phenotype was observed in GA80-GFPa/b zebrafish at 4 dpf. The average percentages of phenotypic fish of the three different classes are indicated in the bargraph. **c** GA80-GFPa/b zebrafish had mostly no circulation at 2 dpf. Red blood cells accumulate due to circulation defects (*arrow*). **d** The average percentages of fish with or without circulation are indicated in the bargraph (at least three independent clutches were analyzed with n ≥14)

Poly-GA has previously been shown to be toxic in neurons and animal models [23, 29]. Expression of GA80-GFP protein was detected by diffuse green GFP expression and as GFP inclusions exclusively in the musculature by 2 and 4 dpf (Fig. 3a), whereas GA2-GFP fish showed only diffuse green fluorescence. Almost all GA80-GFPa expressing fish showed a severe pericardial edema (92.8 ± 2.8%) and only very few fish showed a mild pericardial edema (7.2 ± 2.8%) at 4 dpf. Similarly, the second transgenic line GA80-GFP-b had 97.5 ± 2.6% of the fish with an severe pericardial edema and 2.5 ± 2.6% with an mild pericardial edema at 4 dpf (Fig. 3b).

Additionally, GA80-GFPa/b fish also showed a strongly reduced circulation of red blood cells in 61.5 ± 9.0% (line a) and 21.6 ± 3.9% (line b) of the GFP positive embryos at 2 dpf, respectively. In contrast, wild-type, GA2-GFPa/b as well as ggggcc80-GFP fish did not show any circulation defect of red blood cells at 2 dpf (Fig. 3c,d), suggesting that poly-GA toxicity additionally impaired circulation. The circulation defect did not seem to be mediated by a heart defect since the GA80-GFPa fish had a normal heart beat at 2.5 dpf (data not shown). Phenotypic embryos with a strong edema and lack of circulation died around 5 dpf.

We further examined the length of the spinal motor neuron axons in GA80-GFPa zebrafish, since *C9orf72* repeat expansion carriers can suffer from motor neuron degeneration. No significant differences of the axonal length were observed at 28 hpf (Additional file 2: Figure S2A, B). In addition, there was no apparent difference in the overall neuronal outgrowth and branching at 2 dpf (Additional file 3: Figure S3A, B), indicating expression of neither an expanded GGGGCC repeat nor poly-GA protein affected neuronal outgrowth. Interestingly, aggregates of GA80-GFP were exclusively found in the musculature despite ubiquitous GA80-GFP protein expression in both transgenic GA80-GFP lines at 2 and 4 dpf. Larvae with a severe edema phenotype had more than twice as many inclusions compared to larvae with a mild phenotype at 4 dpf (Additional file 4: Figure S4A), suggesting that these embryos have higher expression levels, leading to higher toxicity and more inclusions. The overall structure of the muscle was not affected as determined by α-actinin staining at 2 dpf (Additional file 4: Figure S4B), suggesting that the GA80-GFP aggregates themselves are not toxic and that muscle defects are not the primary cause of toxicity in our zebrafish model.

Previously, we reported that knockout of the two orthologues of human TDP-43, a key protein in ALS/FTLD, in zebrafish (*tardbp−/−, tardbpl−/−*) showed a circulation phenotype accompanied with mispatterning and increased sprout formation of the vasculature [19]. Since *C9orf72* repeat expansion carriers show cytoplasmic TDP-43 mislocalization and presumably partial TDP-43 loss of function, we analyzed TDP-43 function in the repeat expressing fish. We examined vascular patterning in GA80-GFPa zebrafish by crossing them to Tg(kdrl:HsHRAS-mCherry)^s896^, a reporter line expressing mCherry in all endothelial cells [20]. However, neither vascular mispatterning nor aberrant sprout formation as seen in *tardbp−/−, tardbpl−/−* fish were detected at 2.5 dpf (Additional file 5: Figure S5A, B), ruling out vascular patterning defects as the primary cause of the circulation phenotype. However, endothelial cells appeared thinner and less structured in GA80-GFPa larvae (Additional file 5: Figure S5), most likely due to the lack of perfusion. Reduction of zebrafish Tardbp function leads to an alternative splicing pattern of the second ortholgue Tardbpl, referred to as Tardbpl_tv1, which can fully replace Tardbp function. Impaired Tardbp function (for example by mislocalization) can therefore be monitored by upregulation of the compensatory Tardbpl_tv1 variant. We analyzed the expression levels of Tardbp as well as Tardbpl_tv1 by immunoblotting in GA80-GFPa zebrafish at 2 dpf. Increased levels of Tardbpl_tv1 would indicate partial loss of Tardbp. However, protein levels of Tardbp and Tardbpl_tv1 showed no differences in wild-type and GA80-GFP transgenic fish (Additional file 6: Figure S6), suggesting that the perfusion phenotype is mediated by a TDP-43 independent mechanism.

### Antisense morpholino (AMO) rescues the edema phenotype in GA80-GFP zebrafish

**Fig. 4:**
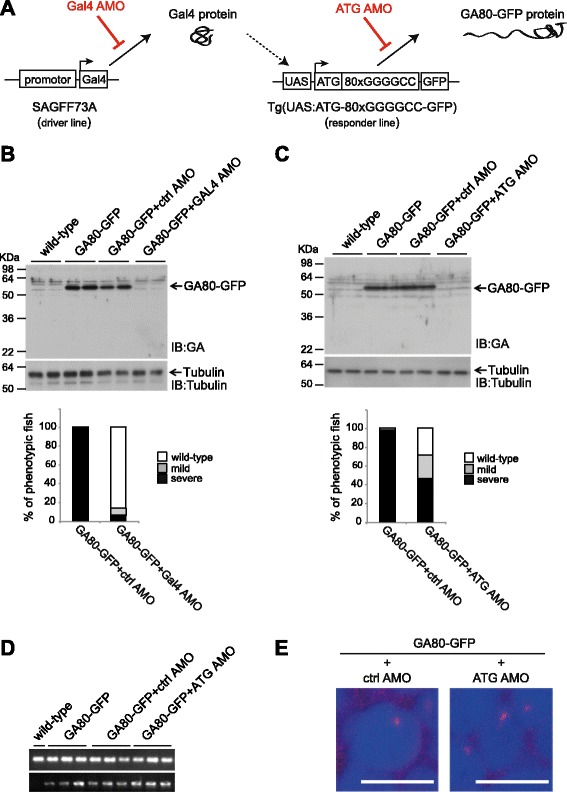
Antisense morpholino rescued the toxic edema phenotype. **a** Representation of process of intervention for each morpholino during the generation of GA80-GFP protein in vivo. **b** GAL4 targeting AMO efficiently blocked Gal4 translation at 2 dpf shown by immunoblotting (*upper panel*). Quantification of the pericardial edema phenotype observed in GA80-GFP with injection of ctrl AMO or GAL4 targeting AMO at 4 dpf are shown as a bar graph (*lower panel*) (*p* < 0,001, 3 independent experiments with 3 clutches *n* ≥ 6 are shown, unpaired *t* test). **c** ATG targeting morpholino efficiently inhibited the ATG dependent translation of poly-GA at 2 dpf (*upper panel*). Quantification of the pericardial edema phenotype observed in GA80-GFP upon injection of ctrl AMO or ATG targeting AMO at 4 dpf shown as a bar graph (*lower panel*) (*p* < 0,005, 3 independent experiments with 3 clutches *n* ≥ 19 are shown, unpaired *t* test). **d** Semi-quantitative RT-PCR analyses of injected embryos at 2 dpf. **e** RNA foci formation was not affected upon injection with ctrl AMO or ATG AMO at 2 dpf. Scale bar 10 μm

Antisense morpholinos (AMO) are a useful tool to suppress the translation of genes of interest. Here, we designed AMOs targeting GAL4 as well as the ATG start codon of the GA80-GFP transgene (Fig. 4a) to further substantiate the correlation of GA80-GFP protein levels and toxicity. Injection of the AMO targeting GAL4 into the fertilized embryos in GA80-GFPa efficiently blocked translation of the transcriptional activator Gal4 and thereby efficiently reduced GA80-GFP protein expression at 2 dpf (Fig. 4b). Although control AMO injected transgenic zebrafish showed mostly the severe edema phenotype (95.8 ± 3.9%) and very few mild edema phenotypes (4.2 ± 3.9%), GAL4 AMO injected fish showed only few severe (4.8 ± 8.2%) and mild (8.6 ± 8.3%) and mostly unaffected zebrafish (86.7 ± 8.3%), indicating that AMO mediated GAL4 inhibition rescued the pericardial edema phenotype at 4 dpf (Fig. 4b).

To further address if toxicity is mediated by the expression of GA80-GFP protein we designed AMO targeting ATG start codon upstream of the GGGGCC repeat (ATG AMO) and aimed at reverting the phenotype by blocking translation of the poly-GA protein in the GA80-GFPa larvae. Upon injection of ATG AMO, GA80-GFP protein expression was severely reduced, as determined by immunoblot at 2 dpf (Fig. 4c). In addition, ATG targeting AMO reduced the severe pericardial edema phenotype in GA80-GFP expressing zebrafish demonstrating that poly-GA protein is a toxic species in vivo at 4 dpf (Fig. 4c.). We next examined the expression level of mRNA as well as RNA foci formation upon ATG targeting AMO injection, since they are blocking translation without changing the mRNA. mRNA expression levels analyzed at 2 dpf by semi-quantitative RT-PCR were unaffected in phenotypic fish upon ATG AMO injection and RNA foci were still detectable (Fig. 4d, e, Additional file 1 Figure S1) indicating that poly-GA was toxic in zebrafish and that the phenotypic rescue is mediated by reduction of GA80-GFP protein levels.

## Discussion

Expansion of a GGGGCC repeat in a non-coding region of *C9orf72* is the most common cause of ALS/FTLD. Recently, RAN translation from the sense and antisense GGGGCC repeat transcript was observed in *C9orf72* repeat expansion carriers generating 5 different DPR species (poly-GA, −GR, −GP, −PA and -PR) [12–14, 30]. These DPRs coaggregate with p62 and form the characteristic star shaped inclusions in *C9orf72* repeat expansion carriers [13].

The relative contribution of RNA and DPR toxicity is still under debate since many conflicting results have been obtained in a variety of different model systems (reviewed in [31]). The GGGGCC repeat RNA forms foci in cells, animal models and patients and has been shown to be able to induce neuronal cell death and to sequester RNA binding proteins [8, 15, 20, 32]. However, there is only a weak correlation between RNA foci and neurodegeneration in patients [33–35]. In zebrafish RNA injection of 8x, 38x, and 72x GGGGCC repeats has been shown to cause RNA foci and cell death by apoptosis in a repeat length dependent manner [9]. This study did not report on RAN translation products upon repeat RNA injections in zebrafish. In line with these studies we observe RNA foci in two independent transgenic ggggcc80-GFP lines and RNA toxicity. In the ggggcc80-GFP fish we were not able to detect GA, GP, and GR species, most likely due to the relatively short repeat length or inefficient or even lack of RAN translation in early zebrafish that preclude detection of DPR species by Western blotting. This is in contrast to fly and mouse models in which repeat expression leads to DPR translation in the absence of a start codon [18, 20]. Whether the mild toxic effects seen in ggggcc80-GFP fish is due to RNA toxicity or low level DPRs remains to be determined. To further address DPR toxicity we focused on poly-GA since it is the most abundant species found in *C9orf72* repeat carriers and induced the neuronal cell death in primary cultured cell model as well as in animal models [16–18, 20]. We generated several transgenic zebrafish lines and demonstrated that poly-GA is toxic in zebrafish. In primary neurons poly-GA toxicity has been attributed to sequestration of Unc119 (a trafficking factor for myristylated proteins), interference with the ubiquitin proteasome system, and endoplasmic reticulum stress [16, 17]. Recently, poly-GR and poly-PR were shown to be the most toxic DPR species in *Drosophila* [18, 36]. Moreover, the arginine-rich DPR species are also toxic in primary neurons, potentially affecting RNA synthesis [18, 19]. Interestingly, DPRs interfere with nucleocytoplasmic shuttling in *Drosophila*, cells, and yeast [21, 22, 24, 25]. Two independently generated BAC transgenic mouse models recapitulate *C9orf72* repeat associated pathology, however they lack neurodegeneration [34, 35]. In contrast, another BAC transgenic mouse model shows neurodegeneration and TDP-43 pathology [37]. Expression of high levels of *C9orf72* repeats by adeno associated virus in mouse brain also generate neurodegeneration and TDP-43 pathology [20]. These differences might reflect that sufficiently high expression levels are required to induce neurodegeneration. It remains unclear which DPR proteins contribute to ALS/FTLD pathogenesis in patients under physiological conditions. There is currently little evidence for a regional correlation of DPR aggregates in humans and neurodegeneration [38]. These animal models will be valuable tools to further dissect the relative contribution and synergistic effects of repeat RNA and DPRs to toxicity.

GA80-GFP fish showed a circulation defect at 2 dpf and a severe pericardial edema phenotype at 4 dpf. Interestingly, double knockout zebrafish (*tardbp−/−, tardbpl−/−*) also showed circulation defects at 2 dpf and vascular mispatterning, resulting in a pericardial edema phenotype reminiscent of the GGGGCC repeat induced phenotype [39]. Considering that partial loss of TDP-43 function could be linked to the pathogenesis of ALS/FTLD-TDP-43, including *C9orf72* repeat expansion carriers [40], we analyzed expression of Tardbp and Tardbpl_tv1 in GA80-GFP fish. However, no apparent changes in Tardbp and Tardbpl_tv1 protein level were observed upon transgene expression, indicating that neither RNA foci nor poly-GA lead to a loss of TDP-43 function in our zebrafish model. We speculate that potentially a common downstream pathway is affected in double knockout zebrafish (*tardbp−/−, tardbpl−/−*) and GA80-GFP zebrafish, resulting in a similar circulation defect. However, since pericardial edemas are a common phenotype, which can be caused by a variety of defects, we cannot exclude the possibility that these similar phenotypes have distinct causes. Unfortunately, the larval lethality precludes analysis of possible neurodegenerative phenotypes of the repeat expressing transgenic zebrafish during adulthood.

GA80-GFP leads to inclusion formation which was restricted to the musculature. Why the musculature was more prone to form inclusions in our model remains speculative. This might be due to higher expression levels of the GA80-GFP protein or driven by cell type specific other coaggregating proteins. There was no correlation of cell death and poly-GA aggregation in zebrafish, since we did not observe any degeneration of muscle cells. Interestingly, there is also no clear correlation between DPR aggregate formation and neuronal loss in *C9orf72* repeat expansion patients, raising the possibility that the aggregates themselves are not the toxic DPR species [26, 38, 41].

## Conclusion

We developed a novel vertebrate animal model for *C9orf72* repeat expansion pathomechanisms and demonstrated that the DPR poly-GA is toxic in vivo. Selective inhibition of poly-GA production by antisense oligonucleotides decreased toxicity. These findings indicate that intervention with DPR expression might be an effective therapeutic strategy for patients with *C9orf72* repeat expansions.

## Methods

### Zebrafish

Zebrafish embryos were kept at 28.5 °C and staged as previously described [35]. AB and TLF were used as the wild-type strains. All experiments were performed in accordance with animal protection standard of Ludwig Maximilians University Munich and approved by the government of upper Bavaria (Regierung von Oberbayern, Munich, Germany).

### Antibodies

Anti-tubulin (Sigma, T6199), anti-GFP (Clontech, 632377), anti-acetylated tubulin (Sigma, T6793), anti-Tardbp (clone 4A12 [19]), anti-Tardbpl_tv1 (clone 16C8 [19]), anti-znp-1 (DSHB), anti-α-actinin (Sigma, A7811), anti-GA (clone 5 F2 [16]), anti-GR (clone 5A2 [9]), anti-mouse IgG, HRP conj. (Promega, W4021), anti-rabbit IgG, HRP conj. (Promega, W4011), Alexa Fluor antibodies (Invitrogen).

### Plasmid construction and generation of transgenic zebrafish

For the construction of the GA2-GFP and GA80-GFP plasmids, a Kozak sequence (GCCGCCACC) was inserted 3′ of the ATG.

For the generation of the GA80-GFP and ggggcc80-GFP plasmids were constructed as described in Additional file 7: Figure S7. pCS2 + eGFP plasmid [13] was PCR amplified by Phusion high fidelity polymerase (New England Biolabs) using the following primers:

- GA80-GFP
  A: pCS2-f1: 5′-ggccgcaGGTGGCGGAGGTGGCGTGAGCAAGGGCGAGGAGC-3′
    pCS2-r1: 5′-gCATGGTGGCGGCCTTGGATCCGGAATTCGAATCGATGGGATCCTGCA-3′
  B: pCS2-f2: 5′- gcaGGTGGCGGAGGTGGCGTGAGCAAGGGCGAGGAGC-3′
    pCS2-r2: 5′- tagCATGGTGGCGGCCTTGGATCCGGAATTCGAATCGATGGGATCCTGCA-3′
  ggggcc80xRNA
  A’: pCS2-f1: 5′- ggccgcaGGTGGCGGAGGTGGCGTGAGCAAGGGCGAGGAGC-3′
    pCS2-r3: 5′- gGGTGGCGGCCTTGGATCCGGAATTCGAATCGATGGGATCCTGCA-3′
  B’ pCS2-f2: 5′- gcaGGTGGCGGAGGTGGCGTGAGCAAGGGCGAGGAGC-3′
    pCS2-r2: 5′- tagCATGGTGGCGGCCTTGGATCCGGAATTCGAATCGATGGGATCCTGCA-3′

After purification of the PCR products generated by A/B or A’/B’, the PCR products were co-incubated at following cycles (94 °C 2 min, 94 °C 30 s, 55 °C 30 s, 72 °C 2 min, 72 °C 10 min, 10 °C 10 min) to produce sticky end fragments digested by NotI/BfaI (New England Biolabs), like for circular plasmid generation. To prepare the gggggcc80-GFP plasmid for the generation of transgenic fish, the pEF-80xGGGGCC plasmid [9] was digested by BamHI and PmeI (New England Biolabs). Subsequently, the 80xGGGGCC fragment was purified and digested by BfaI and NotI. The fragment was then ligated to a PCR amplified pCS2+ plasmid vector backbone.

The pCS2 + ATG-80xGGGGCC-GFP and pCS2 + 80xGGGGCC plasmids were digested by StyI (New England Biolabs) and HpaI (New England Biolabs) and cloned into pT2KXIGdeltaIN plasmid (a gift from K. Kawakami, National Institute of Genetics, Shizuoka, Japan) to generate transgenic zebrafish by TOL2 mediated transposition. Our pT2KXIGdeltaIN plasmid harbored one point mutation in the UAS region that generated a novel StyI recognition site. We therefore had to reintroduce the StyI- StyI digested fragment that was previously lost during the cloning procedure into the pT2 + ATG-80xGGGGCC-GFP/+80xGGGGCC plasmids.

To generate the ATG-2xGGGGCC-GFP plasmid, the primer (ATG-short2:’- AAAAGATCCAAGGCCGCCACCATGCTAGGGGCCGGGGCCGGGGCTCTCAAACT-3′), which includes 2xGGGGCC and a T3 primer were used for amplification of the pCS2 + eGFP plasmid backbone [13]. Subsequently, the PCR product as well as pCS2 + ATG-80xGGGGCC-GFP were digested by StyI and HpaI and ligated to each other. The pT2 + ATG-2XGGGGCC-GFP plasmid was generated accordingly.

To generate the transgenic fish, 10 ng/μl of the corresponding plasmid and 100 ng/μl transposase mRNA were co-injected into 1 cell stage AB embryos.

GA2-GFP and GA80-GFP run at a higher molecular weight than the calculated 31,3 and 41,3 kDa (including the 33aa linker sequences) potentially due to posttranslational modifications. GFP coding sequence without a start codon was used.

### Genotyping the repeat length by PCR

To extract genomic DNA from zebrafish embryos, Proteinase K (Roche) treatment (170 μg/ml, overnight) was performed at 65 °C. Subsequently, Proteinase K was inactivated at 95 °C for 10 min. To amplify the transgene, GFP specific primers were used for genotyping (GFP-3f: 5′- GTGGTGCCCATCCTGGTCGAGCT-3′ and GFP-3r: 5′- AGATCTGAGTCCGGACTTGTACAG-3′).

To confirm the repeat length, Expand Long Template PCR System (Roche) was used with slight modifications: 98 °C 10 min, 97 °C 35 s, 55 °C 2min20s, 68 °C 2min20s, 68 °C 10 min, 55 °C 5 min, 50 °C 5 min, 10 °C. Steps from 2 to 4 were repeated in 49 cycles. [36]. 80rep-1f: 5′-CTAGAGGGTATATAATGGATC-3′ and 80rep-r2: 5′-CTGTGCTGGATATCTGCAGAATT-3′ were used for PCR.

### Whole mount fluorescent in situ hybridization and motor axonal length measurement

Zebrafish embryos (28 hpf) were fixed in 4% paraformaldehyde (PFA) overnight. Protocol modified from Thisse et al. [42]. The embryo was gradually transferred into phosphate buffered saline Tween-20 (PBST), PBST with 30% methanol (MeOH), PBST with 60% MeOH and finally 100% MeOH and kept at −20 °C overnight. Before Proteinase K treatment, embryos were transferred into PBST with 60% MeOH, PBST with 30% MeOH and PBST. After Proteinase K digestion, re-fixation of embryos was performed by PFA for 15 min at room temperature. After washing with PBST for 5 × 10 min, embryos were pre-incubated with hybridization buffer (HYB+) for 1 h at 65 °C. The Cy3(GGCCCC)_4_ probe was synthesized by IDT (Integrated DNA Technologies) as previously described [2] and diluted into 10 ng/μl in HYB+ solution. Embryos were hybridized overnight at 65 °C. Afterwards, they were washed in HYB- for 3 × 30 min, 2 × saline sodium citrate with 0.1% Tween20 (SSCT) for 2 × 15 min, 0.2 × SSCT for 3 × 30 min, PBST 3 × 30 min at 65 °C. After 4,6-diamidin-2-phenylindol (DAPI) staining, embryos were mounted in 1.5% agarose.

DNaseI (Qiagen) or RNaseA (Thermo scientific) treatment of the embryos was performed after Proteinase K treatment, by incubation with the respective enzyme in PBST for 1.5 h at 37 °C prior in situ hybridization.

Measurement of axonal motor neuron axon length was previously described [19].

### Semi-quantitative RT-PCR

The RNeasy kit (Qiagen) was used with on column DNaseI treatment for total RNA isolation. cDNA synthesis was performed with M-MLV reverse transcriptase (Invitrogen) and Random Primer Mix (NEB), followed by a RNaseH (Invitrogen) digest as previously described [16].

### Antisense morpholino (AMO)

Sequences of AMO used in this study:

Control AMO (ctrl AMO) (CCTCTTACCTCAGTTACAATTTATA), GAL4 targeting AMO (Gal4 AMO) (GTTCGATAGAAGACAGTAGCTTCAT) [37], and ATG targeting AMO (ATG AMO) (CCCCTAGCATGGTGGCGGCCTT) were all obtained from Genetools. AMOs were injected into fertilized embryos according to the manufacturer’s instructions. The Gal4 AMO and the ATG AMO were used at 125 μM.

### Western blotting and immunohistochemistry

A standard protocol was used as previously described [16]. To stain the anti-α-actinin or anti-acetylated tubulin, in vivo imaging for mCherry expression to analyze the vasculature, embryos at 2 dpf were fixed by 4% PFA.

### Microscopy

Images were taken with a Cell Observer CSU-X1 (Yokogawa) Spinning Disk (Zeiss), AxioCam MRm (Zeiss) and Evolve 512 (Photometrics) or confocal microscope LSM710 (Zeiss). Brightness and contrast were adjusted using Zen blue or gray (Zeiss) and ImageJ. For in vivo imaging of GFP fluorescence, dechorionated zebrafish were incubated with Tricaine (3-amino benzoic acidethylester) (Sigma) for immobilization. Subsequently, zebrafish were mounted in the Metaphor (low melting temperature) agarose (LONZA).

## Additional files

Additional file 1: Figure S1. RNA foci formation overview. Embryos of the indicated genotypes stained with a Cy3-labeled probe to visualize RNA foci formation by in situ hybridization. Between 13–33 cells per field of view showed RNA foci in the GA80-GFP larvae. All images were taken without DAPI fluorescence. Scale bar 10 μm. (PDF 2702 kb)
Additional file 2: Figure S2. Spinal motor neuron axonal outgrowth is not affected. (A) Spinal motor neuron axon of GA80-GFP fish (Gal4 driver + UAS:ATG-80xGGGGCC-GFP responder) and the GFP negative siblings (Gal4 driver or UAS:ATG-80xGGGGCC-GFP alone, or wild-type) at 28 hpf. (B) Length of outgrowing spinal motor neuron axons measured from the exit point of the spinal cord to the tip of the growth cone in the 5 somites anterior of the end of the yolk expansion at 28 hpf (indicated by the numbers 1–5). Embryos are sorted by the genotypes wild-type, driver, responder, and driver + responder. Statistical analyses was performed in indicated genotypes. Scale bar 20 μm. Mean ± SD. (PDF 4905 kb)
Additional file 3: Figure S3. Overall neuronal outgrowth is not affected. Overall neuronal outgrowth was analyzed in embryos stained with an antibody against acetylated tubulin at 2 dpf. Siblings of GA80-GFPa zebrafish expressing GFP (A) or not expressing GFP (B). Scale bar 100 μm. (PDF 6302 kb)
Additional file 4: Figure S4. Muscle patterning is not affected. (A) Quantification of GFP inclusions in GA80-GFPa and GA80-GFPb larvae subdivided into mild and strong edema phenotypes at 4 dpf. (n = 4 per subgroup, mean ± SD). Amount of inclusions from GA80-GFP line a and b with mild and strong phenotypes were not significantly different (paired *t*-test). Inclusions were exclusively detected in the musculature in both lines. (B) The overall structure of the muscle was analyzed by α-actinin staining at 2 dpf in a GFP negative GA80-GFP embryo and (B) GFP positive sibling. Scale bar 20 μm. (PDF 3472 kb)
Additional file 5: Figure S5. Vascular patterning is not affected. The vasculature was analyzed by incrossing with Tg(kdrl:HsHRAS-mCherry)^s896^ into the GA80-GFP expressing lines. mCherry expressed from the Tg(kdrl:HsHRAS-mCherry)^s896^ transgene is shown in Ga80-GFP-a transgenic zebrafish not expressing GFP (A) and siblings expressing GFP (B) at 2.5 dpf. Scale bar 20 μm. (PDF 4003 kb)
Additional file 6: Figure S6. Tardbp function is not impaired in repeat expressing fish. (A) GA80-GFPa zebrafish expressing GFP and (B) siblings not expressing GFP. Western blot analysis of 2 dpf old embryos with antibodies as indicated. Tardbp/Tardbpl_tv1 bands indicated by arrow heads. (PDF 3086 kb)
Additional file 7: Figure S7. Construction of repeat expressing plasmid in zebrafish. A representative scheme to generate the 80xggggcc repeat containing plasmid. (PDF 1457 kb)
