# Supplementary figures and images for: Glycine-alanine dipeptide repeat protein contributes to toxicity in a zebrafish model of C9orf72 associated neurodegeneration
Source: Mol Neurodegener. 2017 Jan 14;12:6. doi: 10.1186/s13024-016-0146-8 (PMC5237533; doi:10.1186/s13024-016-0146-8)

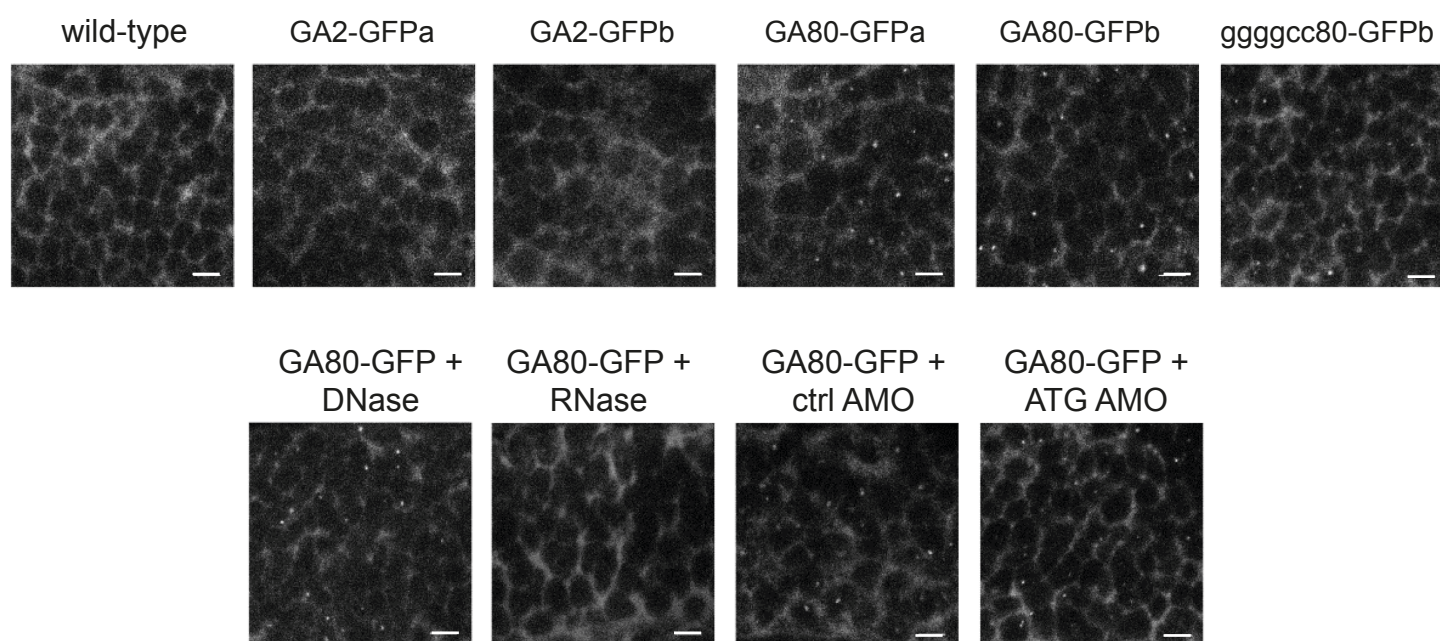

Supplement: Additional file 1: Figure S1. — RNA foci formation overview. Embryos of the indicated genotypes stained with a Cy3-labeled probe to visualize RNA foci formation by in situ hybridization. Between 13–33 cells per field of view showed RNA foci in the GA80-GFP larvae. All images were taken without DAPI fluorescence. Scale bar 10 μm. (PDF 2702 kb) [file 13024_2016_146_MOESM1_ESM.pdf]

**A**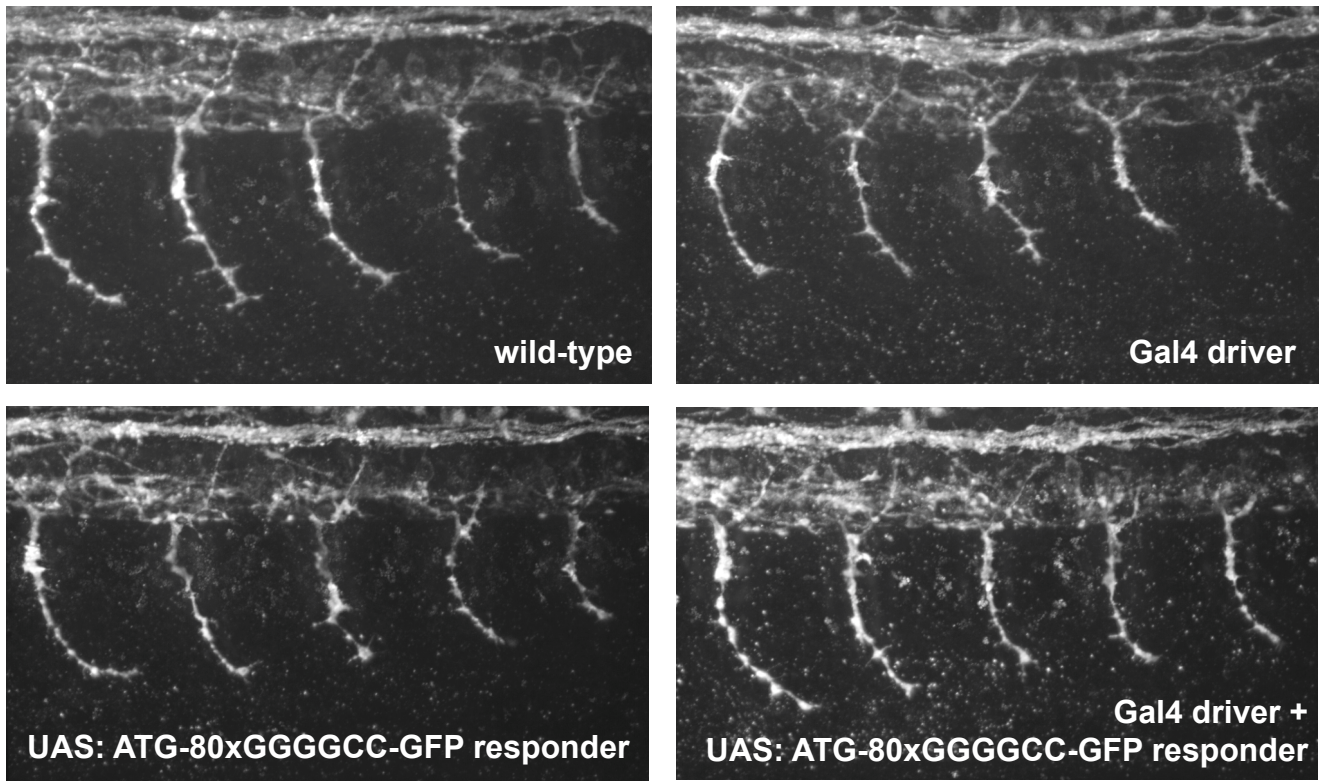**B**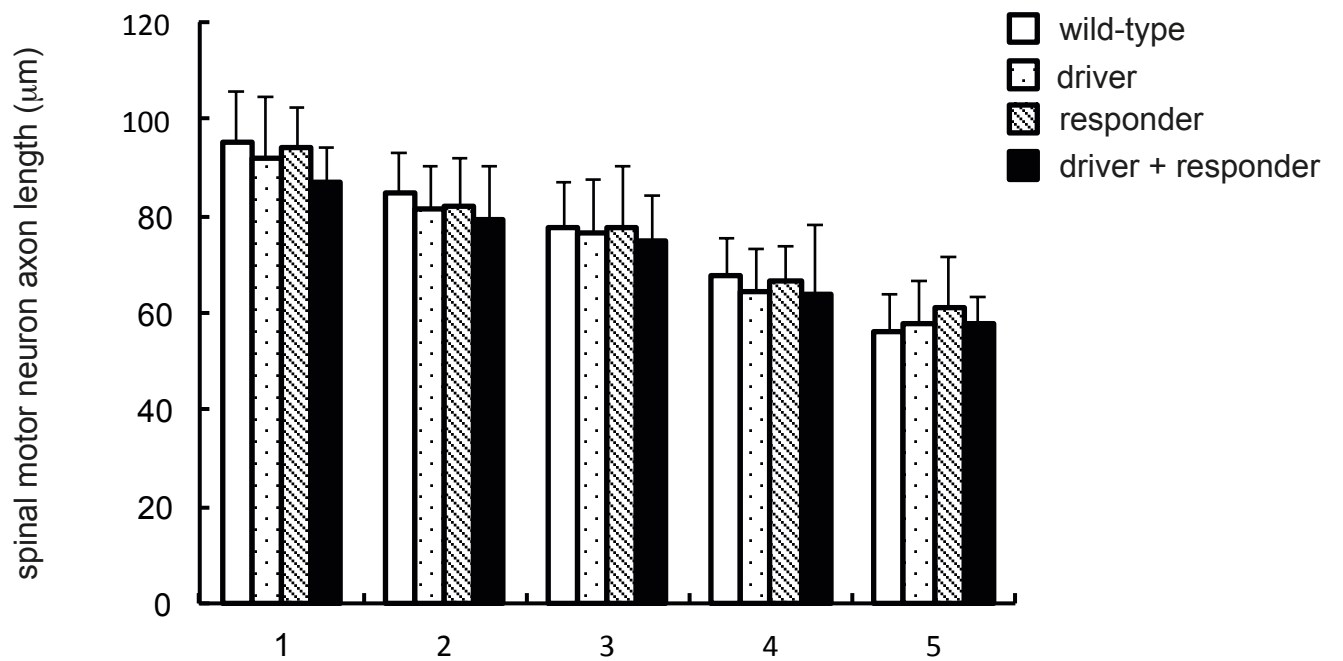

Supplement: Additional file 2: Figure S2. — Spinal motor neuron axonal outgrowth is not affected. (A) Spinal motor neuron axon of GA80-GFP fish (Gal4 driver + UAS:ATG-80xGGGGCC-GFP responder) and the GFP negative siblings (Gal4 driver or UAS:ATG-80xGGGGCC-GFP alone, or wild-type) at 28 hpf. (B) Length of outgrowing spinal motor neuron axons measured from the exit point of the spinal cord to the tip of the growth cone in the 5 somites anterior of the end of the yolk expansion at 28 hpf (indicated by the numbers 1–5). Embryos are sorted by the genotypes wild-type, driver, responder, and driver + responder. Statistical analyses was performed in indicated genotypes. Scale bar 20 μm. Mean ± SD. (PDF 4905 kb) [file 13024_2016_146_MOESM2_ESM.pdf]

**A**

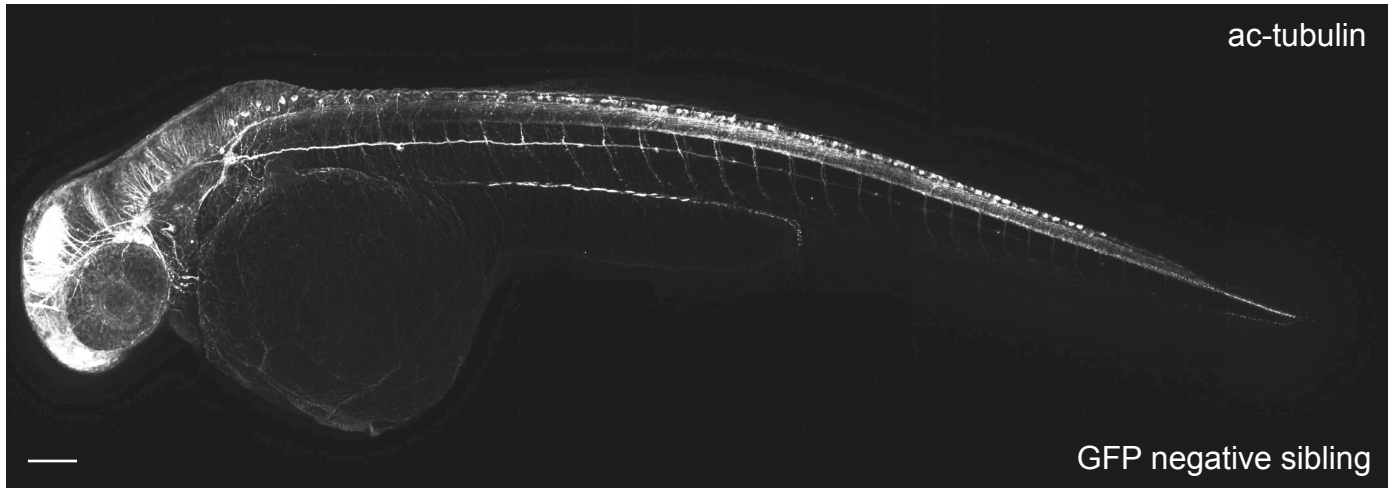

**B**

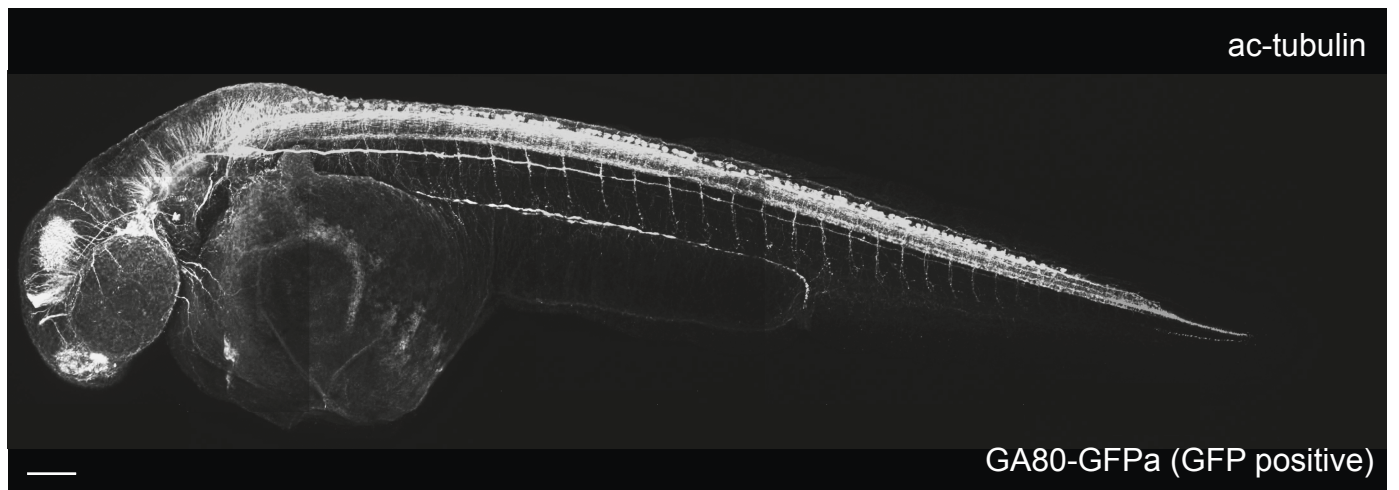

Supplement: Additional file 3: Figure S3. — Overall neuronal outgrowth is not affected. Overall neuronal outgrowth was analyzed in embryos stained with an antibody against acetylated tubulin at 2 dpf. Siblings of GA80-GFPa zebrafish expressing GFP (A) or not expressing GFP (B). Scale bar 100 μm. (PDF 6302 kb) [file 13024_2016_146_MOESM3_ESM.pdf]

**A**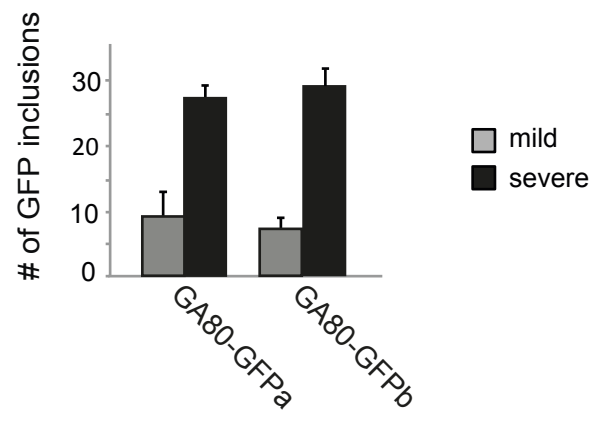**B**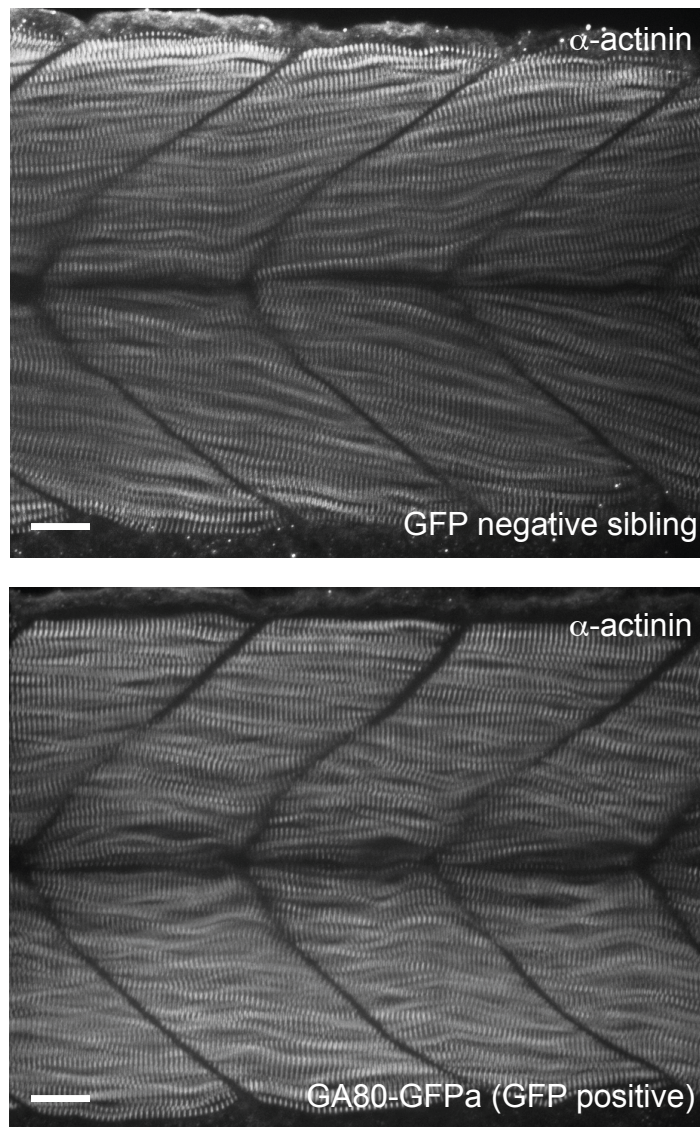

Supplement: Additional file 4: Figure S4. — Muscle patterning is not affected. (A) Quantification of GFP inclusions in GA80-GFPa and GA80-GFPb larvae subdivided into mild and strong edema phenotypes at 4 dpf. (n = 4 per subgroup, mean ± SD). Amount of inclusions from GA80-GFP line a and b with mild and strong phenotypes were not significantly different (paired t-test). Inclusions were exclusively detected in the musculature in both lines. (B) The overall structure of the muscle was analyzed by α-actinin staining at 2 dpf in a GFP negative GA80-GFP embryo and (B) GFP positive sibling. Scale bar 20 μm. (PDF 3472 kb) [file 13024_2016_146_MOESM4_ESM.pdf]

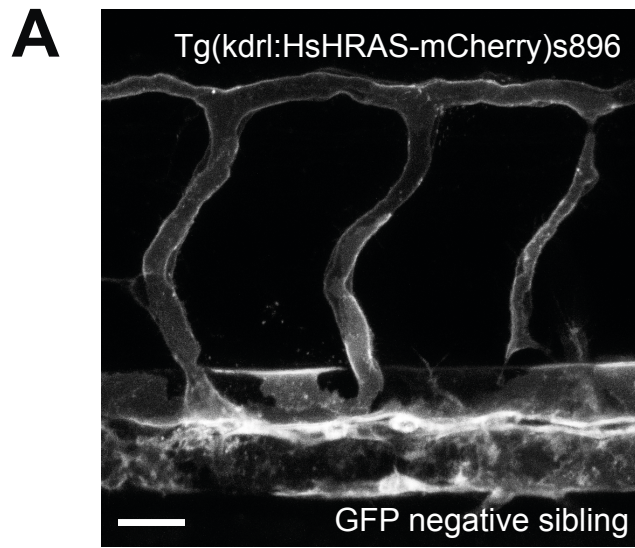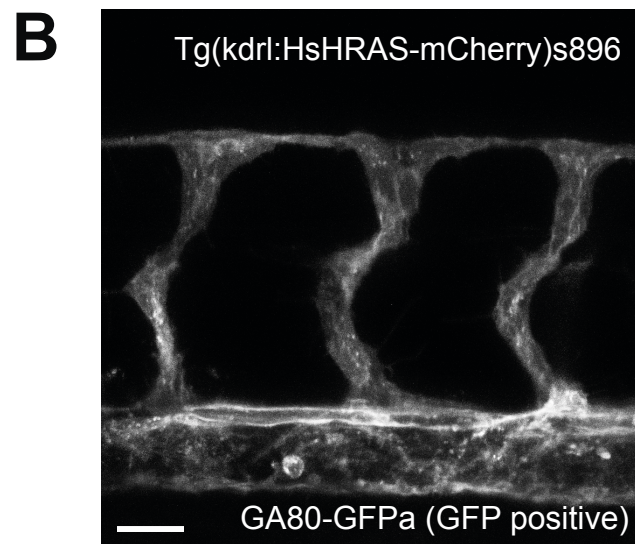

Supplement: Additional file 5: Figure S5. — Vascular patterning is not affected. The vasculature was analyzed by incrossing with Tg(kdrl:HsHRAS-mCherry)s896 into the GA80-GFP expressing lines. mCherry expressed from the Tg(kdrl:HsHRAS-mCherry)s896 transgene is shown in Ga80-GFP-a transgenic zebrafish not expressing GFP (A) and siblings expressing GFP (B) at 2.5 dpf. Scale bar 20 μm. (PDF 4003 kb) [file 13024_2016_146_MOESM5_ESM.pdf]

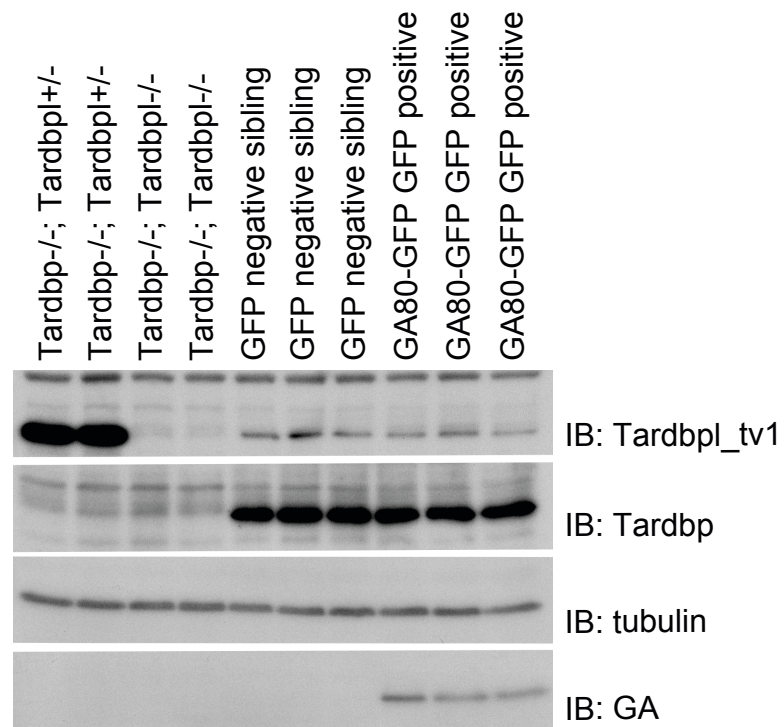

Supplement: Additional file 6: Figure S6. — Tardbp function is not impaired in repeat expressing fish. (A) GA80-GFPa zebrafish expressing GFP and (B) siblings not expressing GFP. Western blot analysis of 2 dpf old embryos with antibodies as indicated. Tardbp/Tardbpl_tv1 bands indicated by arrow heads. (PDF 3086 kb) [file 13024_2016_146_MOESM6_ESM.pdf]
