# Supplementary material for: Glycine-alanine dipeptide repeat protein contributes to toxicity in a zebrafish model of C9orf72 associated neurodegeneration
Source: Mol Neurodegener. 2017 Jan 14;12:6. doi: 10.1186/s13024-016-0146-8 (PMC5237533; doi:10.1186/s13024-016-0146-8)
Supplement: Additional file 7: Figure S7. — Construction of repeat expressing plasmid in zebrafish. A representative scheme to generate the 80xggggcc repeat containing plasmid. (PDF 1457 kb) [file 13024_2016_146_MOESM7_ESM.pdf]

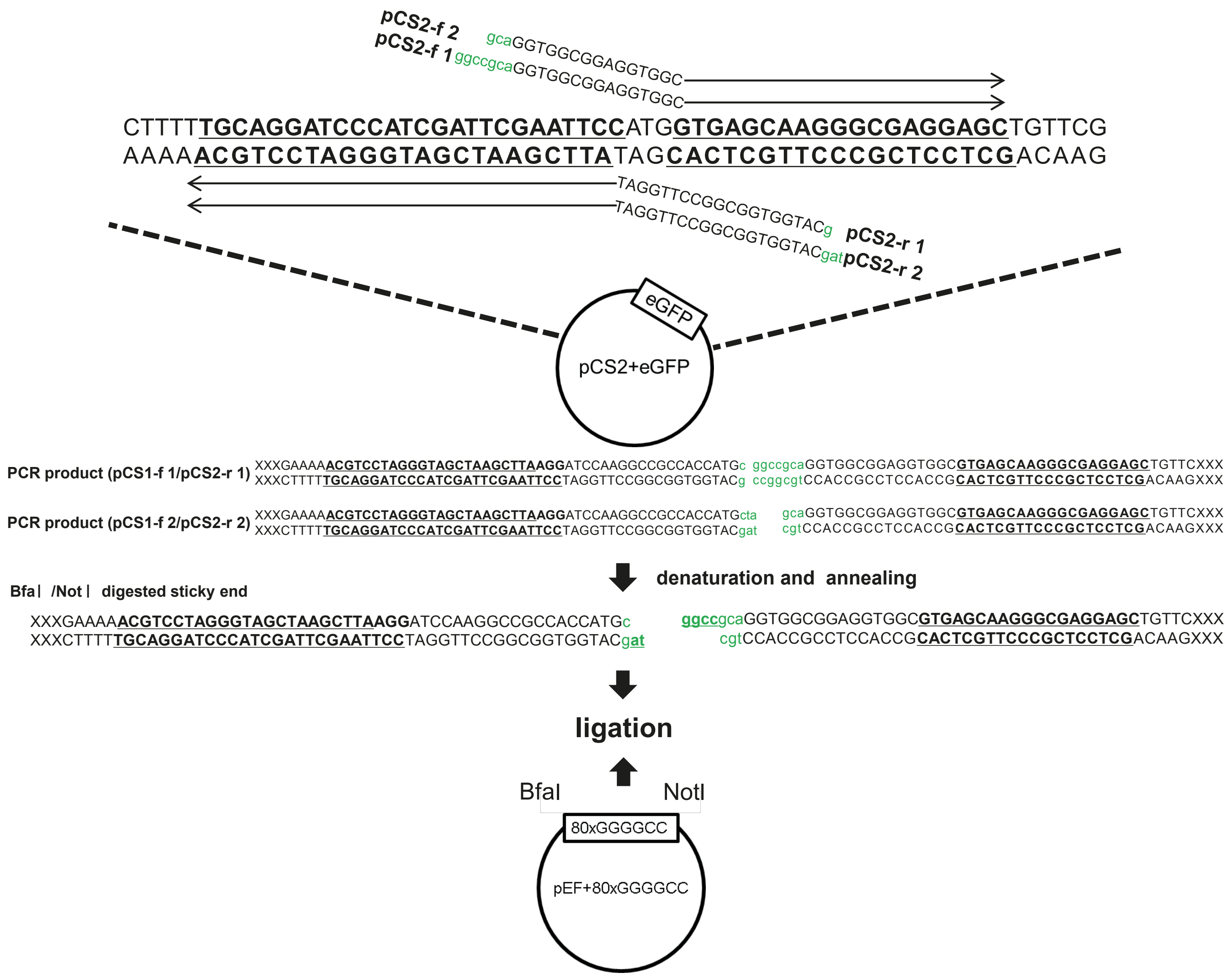

Ohki et al., sFig.7
